# Is combined extra-hepatic bile-duct resection justified for advanced gallbladder carcinoma?

**DOI:** 10.1093/gastro/goz018

**Published:** 2019-05-25

**Authors:** Jun-Ke Wang, Wen-Jie Ma, Zhen-Ru Wu, Qin Yang, Hai-Jie Hu, Fei Liu, Fu-Yu Li

**Affiliations:** 1 Department of Biliary Surgery, West China Hospital, Sichuan University, Chengdu, Sichuan, P. R. China; 2 Laboratory of Pathology, West China Hospital, Sichuan University, Chengdu, Sichuan, P. R. China

**Keywords:** gallbladder carcinoma, curative surgery, extra-hepatic bile-duct resection, overall survival

## Abstract

**Background:**

Whether the extra-hepatic bile duct (EHBD) should be routinely resected for gallbladder carcinoma (GBC) remains controversial. The current study aimed to determine the clinical impact of combined EHBD resection during curative surgery for advanced GBC.

**Methods:**

In total, 213 patients who underwent curative surgery for T2, T3 or T4 GBC were enrolled. The clinicopathological features were compared between the patients treated with EHBD resection and those without EHBD resection. Meanwhile, univariable and multivariable Cox-proportional hazards regression models were used to identify risk factors for overall survival (OS).

**Results:**

Among the 213 patients identified, 87 (40.8%) underwent combined EHBD resection. Compared with patients without EHBD resection, patients with EHBD resection suffered more post-operative complications (33.3% vs. 21.4%, *P* = 0.046). However, the median OS of the EHBD resection group was longer than that of the non-EHBD resection group (25 vs. 11 months, *P* = 0.008). Subgroup analyses were also performed according to tumor (T) category and lymph-node metastasis. The median OS was significantly longer in the EHBD resection group than in the non-EHBD resection group for patients with T3 lesion (15 vs. 7 months, *P* = 0.002), T4 lesion (11 vs. 6 months, *P* = 0.021) or lymph-node metastasis (12 vs. 7 months, *P* < 0.001). No survival benefit of EHBD resection was observed in GBC patients with T2 lesion or without lymph-node metastasis. T category, lymph-node metastasis, margin status, pre-operative CA19-9 level and EHBD resection were identified as independent prognostic factors for OS of patients with advanced GBC (all *P* values <0.05).

**Conclusions** EHBD resection can independently affect the OS in advanced GBC. For GBC patients with T3 lesion, T4 lesion and lymph-node metastasis, combined EHBD resection is justified and may improve OS.

## Introduction

Gallbladder carcinoma (GBC) is the most common biliary tract malignancy and the fifth most common gastrointestinal cancer [1, 2]. Owing to nonspecific clinical manifestations, most patients are diagnosed at advanced tumor stages with a dismal prognosis [3]. Undergoing curative surgery is the only chance to achieve long-term survival for GBC patients. For Tis/T1 GBC, simple cholecystectomy has been demonstrated as a curative procedure [4, 5]. For advanced GBC with T1 or higher-level lesions, radical cholecystectomy, including liver and gallbladder resection combined with regional lymph-node dissection, has been widely performed to achieve curative resection [6]. However, the optimal extent of the surgical procedures, such as the extent of hepatic resection and lymph-node dissection as well as the necessity for routine extra-hepatic bile-duct (EHBD) resection, are controversial [7–12].

Bile-duct infiltration represents an advanced stage of GBC and EHBD resection offers the only chance for complete tumor eradication. Several investigators suggested routine resection of the EHBD for complete dissection of lymph nodes and occult tumor cells [7, 8, 13], whereas others argue that EHBD resection has no survival benefit but increases post-operative complications, such as bile leakage [9, 14–19]. Furthermore, tumor (T) category and lymph-node metastasis have been considered the most significant prognostic factors influencing the overall survival (OS) of GBC patients. However, when analysing the prognostic effect of EHBD resection on GBC, few previous studies had taken these two significant factors into consideration. The operative indications of combined EHBD resection for advanced GBC should be carefully re-evaluated.

In the present study, we compared the clinicopathological factors of advanced GBC patients based on EHBD resection to evaluate the clinical impact of combined EHBD resection on advanced GBC. Furthermore, subgroup analyses of the OS were also performed according to the T category and presence of lymph-node metastasis.

## Patients and methods

### Patient selection

Between January 2007 and January 2016, 489 consecutive GBC patients underwent surgery at West China Hospital of Sichuan University (China). Of these patients, 262 underwent palliative surgery or had unresectable tumor, whereas 227 patients underwent curative surgery. R0 resection means a complete surgical resection with no microscopic residual tumor, whereas R1 resection means a complete surgical resection with no grossly visual tumor; the curative surgery, including R0 and R1 resection, was defined grossly as no residual tumor. Of the 227 patients, 14 with Tis/T1 GBC, according to the 7th edition of TNM classification of the American Joint Committee on Cancer (AJCC), were excluded from the present study. Ultimately, 213 GBC patients with T2, T3 or T4 lesions who underwent curative surgery were enrolled in this study. Their clinicopathological features, including pre-operative, intra-operative and post-operative data, were reviewed. Of the 213 patients included in our study, 87 underwent EHBD resection, whereas 126 did not. No patients accepted neoadjuvant chemotherapy before the curative surgery. All patients were pathologically diagnosed with GBC after surgery.

### Surgical procedure

All 213 patients underwent radical cholecystectomy, which involved gallbladder resection, some extent of liver resection and lymph-node dissection. For patients who were incidentally diagnosed with GBC after simple cholecystectomy for benign diseases, secondary radical surgery was performed for curative intent. The surgical procedure depended on the extent of the tumor. The extent of liver resection was variable, ranging from wedge resection of the gallbladder bed (*n* = 36) and segment IVb and V resection (*n* = 123) to right hemihepatectomy (*n* = 43) and right trisegmentectomy (*n* = 11). Lymph-node dissection was performed in all patients, either D1 (*n* = 139) or D2 (*n* = 74) dissection and at least one lymph node was retrieved. D1 dissection involved lymph nodes around the hepatoduodenal ligament (along the cystic duct, bile duct, portal vein and hilum of the liver), whereas D2 dissection involved lymph nodes around the pancreatic head, duodenum and celiac artery. Patients with jaundice, direct EHBD involvement and positive cystic duct margin were considered potential candidates for combined EHBD resection. When the tumor invaded the adjacent organs, including the stomach and duodenum (*n* = 6), and the colon (*n* = 9), these organs were also resected for curative intent. Pancreaticoduodenectomy was performed in 12 patients due to the following reasons: lower bile-duct involvement (*n* = 5), pancreatic infiltration (*n* = 2) and peripancreatic lymph-node metastasis (*n* = 5). Portal-vein resection and reconstruction (*n* = 13) and hepatic artery resection and reconstruction (*n* = 16) were performed if necessary. Post-operative complications by new classification system [20] were observed in 56 patients: bile leakage (*n* = 17), hepatic failure (*n* = 13), lung infection (*n* = 6), hemorrhage (*n* = 6), peritoneal cavity infection (*n* = 5), pancreatic fistula (*n* = 4), sepsis (*n* = 2), renal failure (*n* = 2) and acute cardiac failure (*n* = 1). Five patients died within 60 days after radical surgery for the following reasons: intra-abdominal bleeding (*n* = 2), liver failure (*n* = 2) and lung infection (*n* = 1).

### Follow-up

Patients were strictly followed up by outpatient check-ups and telephone interview after surgery. General examinations such as liver functions, tumor markers and abdominal ultrasound were conducted every 2–3 months during the first year after surgery, then 3–6 months from the second year. Abdominal computed tomography or magnetic resonance imaging was further conducted if patients were suspected of having tumor recurrence.

### Statistical analysis

Statistical analysis was performed by IBM SPSS version 20.0 (IBM SPSS, Chicago, IL, USA). Continuous data are presented as medians (ranges) and categorical data are presented as numbers (percentages). Comparisons between groups were performed using *χ^2^* test, Fisher’s exact probability test or Mann–Whitney’s *U* test, where appropriate. OS, defined as the period from the date of surgery to death or the last follow-up, was estimated by using the Kaplan–Meier method and differences in survival were examined by using log-rank test. A multivariate Cox-proportional hazards model was used to identify independent prognostic factors with *P* < 0.05 in univariate analysis. The results of the multivariable analysis are presented with hazard ratios (HRs) and corresponding 95% confidence intervals (CIs). *P* < 0.05 was deemed statistically significant.

## Results

### Baseline characteristics

Of the 213 patients identified, the median age was 63 years (range, 36–85 years); 139 (65.3%) were women. The median pre-operative levels of total bilirubin (TB), alanine aminotransferase (ALT) and aspartate transaminase (AST) were 12 µmol/L (range, 4.7–287.3 µmol/L), 23 IU/L (range, 6–842 IU/L) and 24 IU/L (range, 13–850 IU/L), respectively. The pre-operative levels of median CA19-9 and CA125 levels were 18.3 U/mL (range, 0.1 to >1000 U/mL) and 19.6 U/mL (range, 2.1–1657 U/mL), respectively. Among 213 GBC patients, 90 (42.3%), 91 (42.7%) and 32 (15.0%) had T2, T3 and T4 lesions, respectively; 68 (31.9%), 92 (43.2%) and 53 (24.9%) had stage II, III and IV diseases. Overall, 104 (48.8%) patients had lymph-node metastasis. Of the 213 patients, 178 (83.6%) underwent an R0 resection.

### Comparison of patient characteristics between EHBD resection and non-EHBD resection groups

The clinicopathological features of the 213 patients, grouped by EHBD resection, are summarized in Table 1. The two groups had comparable demographics, such as age and sex distribution. Several differences in disease characteristics between the two groups were identified. The pre-operative TB level in the EHBD resection group was substantially higher than that in the non-EHBD resection group (13.0 vs. 11.6 µmol/L, *P* = 0.031). Similarly, more patients in the EHBD resection group received pre-operative biliary drainage with percutaneous transhepatic biliary drainage (PTCD) than those in the non-EHBD resection group (12.6% vs. 2.4%, *P* = 0.003). With respect to the pathologic outcomes, the two groups had comparable rates of R0 resection (78.2% vs. 87.3%, *P* = 0.077) and lymph-node metastasis (52.9% vs. 46.0%, *P* = 0.326). However, patients in the EHBD resection group suffered more perineural invasion than those in the non-EHBD resection group (28.7% vs. 13.5%, *P* = 0.006). For the number of lymph nodes, the EHBD resection group had a higher median number of lymph nodes retrieved (6 vs. 4, *P* < 0.001). In the patients with lymph-node metastasis, the number of positive lymph nodes in the EHBD group was larger than that in the non-EHBD resection group (3 vs. 2, *P* = 0.006). In total, the two groups had comparable death rates (2.3% vs. 2.4%, *P* = 0.969); however, the EHBD resection group suffered more post-operative complications (33.3% vs. 21.4%, *P* = 0.046). In particular, the frequency of bile leakage in the EHBD resection group was higher than that in the non-EHBD resection group (9.2% vs. 4.0%, *P* = 0.117), but the difference was not statistically significant.

**Table 1.** Clinical characteristics of advanced GBC patients grouped by EHBD resection

Variable	Before propensity score matching			After propensity score matching		
	EHBD resection (*n* = 87)	Non-EHBD resection (*n* = 126)	*P*	EHBD resection (*n* = 87)	Non-EHBD resection (*n* = 72)	*P*
Age, years [range]	60 [37–85]	64 [36–83]	0.246	60 [37–85]	65 [36–83]	0.303
Sex						
Female	60 (69.0%)	79 (62.7%)	0.345	60 (69.0%)	50 (69.4%)	0.948
Male	27 (31.0%)	47 (37.3%)		27 (31.0%)	22 (30.6%)	
TB, µmol/L [range]	13.0 [5.6–287.3]	11.6 [4.7–196.0]	0.031	13.0 [5.6–287.3]	9.3 [4.7–141.7]	< 0.010
ALT, IU/L [range]	24 [6–842]	21 [10–604]	0.228	24 [6–842]	17 [10–172]	0.016
AST, IU/L [range]	25 [13–850]	24 [13–243]	0.555	25 [13–850]	22 [15–197]	0.212
ALB, g/L [range]	41.8 [26.4–50.2]	40.9 [29.5–52.4]	0.256	41.8 [26.4–50.2]	41.0 [31.0–51.4]	0.639
CA19-9, U/mL [range]	21.3 [0.2–> 1000]	18.3 [0.1–> 1000]	0.869	21.3 [0.2–> 1000]	15.9 [0.1–> 1000]	0.599
CA125, U/mL [range]	19.6 [2.1–1657]	20.0 [2.5–1342]	0.753	19.6 [2.1–1657]	20.1 [2.5–401]	0.673
Cholelithiasis	41 (47.1%)	50 (39.7%)	0.280	41 (47.1%)	28 (38.9%)	0.297
Tumor location on the gallbladder						
Fundus	36 (41.4%)	73 (57.9%)	0.066	36 (41.4%)	44 (61.1%)	0.103
Body	30 (34.5%)	37 (29.4%)		30 (34.5%)	16 (22.2%)	
Neck	13 (14.9%)	9 (7.1%)		13 (14.9%)	7 (9.7%)	
Unclear	8 (9.2%)	7 (5.6%)		8 (9.2%)	5 (7.0%)	
Pre-operative biliary drainage						
ENBD	6 (6.9%)	4 (3.2%)	0.207	6 (6.9%)	3 (4.2%)	0.458
PTCD	11 (12.6%)	3 (2.4%)	0.003	11 (12.6%)	0 (0%)	–
Surgical procedures						
Extent of hepatectomy						
Wedge resection	11 (12.6%)	25 (19.8%)	0.189	11 (12.6%)	13 (18.1%)	0.112
Segment IVb and V resection	56 (64.4%)	67(53.2%)		56 (64.4%)	34 (47.2%)	
Right hemihepatectomy	14 (16.1%)	29 (23.0%)		14 (16.1%)	21 (29.1%)	
Right trisegmentectomy	6 (6.9%)	5 (4.0%)		6 (6.9%)	4 (5.6%)	
Lymph-node dissection	87 (100%)	126 (100%)	–	87 (100%)	72 (100%)	–
Pancreatoduodenectomy	12 (13.8%)	0 (0%)	–	12 (13.8%)	0 (0%)	–
Hepatic artery reconstruction	7 (8.0%)	9 (7.1%)	0.806	7 (8.0%)	7 (9.7%)	0.710
Portal-vein reconstruction	7 (8.0%)	6 (4.8%)	0.325	7 (8.0%)	4 (5.6%)	0.538
Adjacent organs resection						
Colon	5 (5.7%)	4 (3.2%)	0.568	5 (5.7%)	2 (2.8%)	0.364
Stomach, duodenum	4 (4.6%)	2 (1.6%)	0.377	4 (4.6%)	2 (2.8%)	0.549
Estimated blood loss, mL [range]	600 [200–3000]	500 [200–2600]	0.877	600 [200–3000]	400 [200–2300]	0.531
Depth of tumor invasion						
T2	38 (43.7%)	52 (41.3%)	0.935	38 (43.7%)	29 (40.3%)	0.745
T3	36 (41.4%)	55 (43.6%)		36 (41.4%)	29 (40.3%)	
T4	13 (14.9%)	19 (15.1%)		13 (14.9%)	14 (19.4%)	
Lymph-node metastasis						
N0	41 (47.1%)	68 (54.0%)	0.326	41 (47.1%)	41 (56.9%)	0.218
N+	46 (52.9%)	58 (46.0%)		46 (52.9%)	31 (43.1%)	
No. of retrieved LNs [range]	6 [1–18]	4 [1–15]	< 0.001	6 [1–18]	4 [1–15]	< 0.001
No. of positive LNs [range]	3 [1–14]^a^	2 [1–12]^b^	0.006	3 [1–14]^c^	2 [1–12]^d^	0.027
7th AJCC stage						
II	26 (29.9%)	42 (33.3%)	0.843	26 (29.9%)	27 (37.5%)	0.581
III	38 (43.7%)	54 (42.9%)		38 (43.7%)	27 (37.5%)	
IV	23 (26.4%)	30 (23.8%)		23 (26.4%)	18 (25.0%)	
Margin status						
R0	68 (78.2%)	110 (87.3%)	0.077	68 (78.2%)	58 (80.6%)	0.711
R1	19 (21.8%)	16 (12.7%)		19 (21.8%)	14 (19.4%)	
Tumor differentiation						
Well/moderately	34 (39.1%)	51 (40.5%)	0.838	34 (39.1%)	23 (31.9%)	0.350
Poorly	53 (60.9%)	75 (59.5%)		53 (60.9%)	49 (68.1%)	
Perineural invasion	25 (28.7%)	17 (13.5%)	0.006	25 (28.7%)	10 (13.9%)	0.025
Vascular invasion	19 (21.8%)	23 (18.3%)	0.518	19 (21.8%)	10 (13.9%)	0.196
Mortality	2 (2.3%)	3 (2.4%)	0.969	2 (2.3%)	0 (0%)	–
Post-operative complications	29 (33.3%)	27 (21.4%)	0.046	29 (33.3%)	11 (15.3%)	0.008
Bile leakage	8 (9.2%)	5 (4.0%)	0.117	8 (9.2%)	4 (5.6%)	0.387
Other causes^e^	21 (24.1%)	22 (17.4%)	–	21 (24.1%)	7 (9.7%)	–
Post-operative stay, days [range]	7 [3–47]	7.5 [3–44]	0.220	7 [3–47]	8 [4–44]	0.083
Post-operative chemotherapy	20 (23.0%)	31 (24.6%)	0.786	20 (23.0%)	17 (23.6%)	0.926

GBC, gallbladder carcinoma; EHBD, extra-hepatic bile duct; TB, total bilirubin; ALT, alanine aminotransferase; AST, aspartate transaminase; ALB, albumin; ENBD, endoscopic biliary drainage; PTCD, percutaneous transhepatic biliary drainage; No., number; LNs, lymph nodes; AJCC, American Joint Committee on Cancer.

^a, b, c, d^ The numbers of patients with positive LNs were 46, 58, 46, and 31, respectively.

^e^ Other causes of post-operative complications include hepatic failure, lung infection, hemorrhage, peritoneal cavity infection, pancreatic fistula, sepsis, renal failure, and acute cardiac failure.

### Survival outcomes

**Figure 1.**
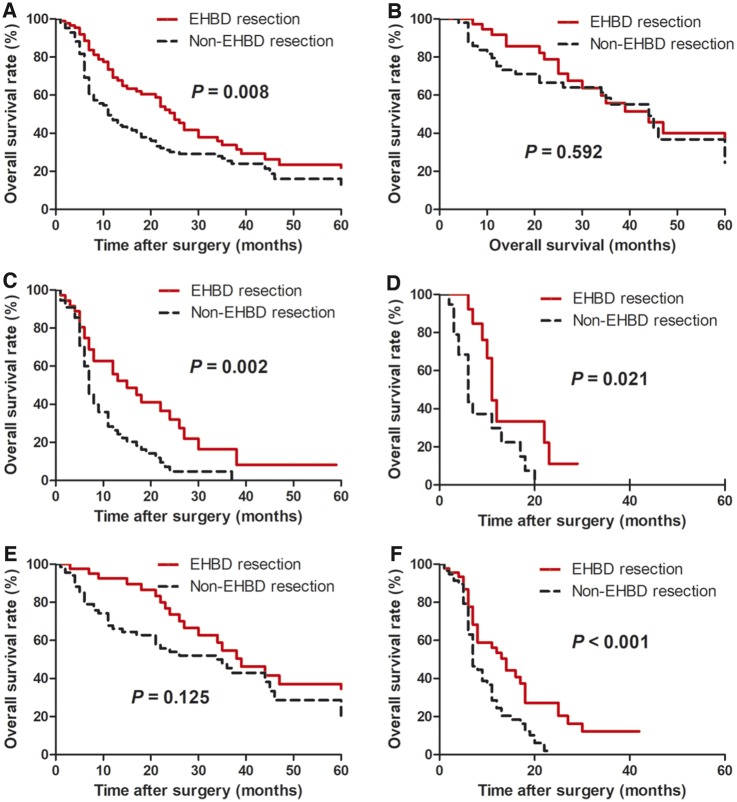
Kaplan–Meier OS analysis of patients with advanced GBC undergoing EHBD resection before propensity score matching. (**A**) The entire cohort; (**B**) the patients with T2 lesion; (**C**) the patients with T3 lesion; (**D**) the patients with T4 lesion; (**E**) the patients without lymph-node metastasis; (**F**) the patients with lymph-node metastasis. OS, overall survival; GBC, gallbladder carcinoma; EHBD, extra-hepatic bile duct.

The median survival time of the entire cohort was 17 months (range, 1–71 months) and the 1-, 3-, and 5-year survival rates were estimated as 59%, 29%, and 20%, respectively. In total, patients who underwent EHBD resection had a longer median OS than patients without EHBD resection (25 vs. 11 months, *P* = 0.008; Figure 1A). Subgroup analyses were performed according to the most significant prognostic factors: T category (T2, T3 or T4) and the presence of lymph-node metastasis. The EHBD resection group achieved longer median OS than the non-EHBD resection group for the patients with T3 lesion (15 vs. 7 months, *P* = 0.002; Figure 1C), T4 lesion (11 vs. 6 months, *P* = 0.021; Figure 1D) and lymph-node metastasis (12 vs. 7 months, *P* < 0.001, Figure 1F). However, no survival benefit of EHBD resection was observed in GBC patients with T2 lesion (44 vs. 44 months, *P* = 0.592; Figure 1B) or without lymph-node metastasis (39 vs. 35 months, *P* = 0.125; Figure 1E).

### Prognostic factors of OS

To identify the independent prognostic factors for advanced GBC, various clinicopathological factors were analysed by using univariate and multivariate Cox-proportional hazards regression models (Table 2). The univariate analysis results showed that pre-operative CA19-9 level (*P* < 0.001), T category (*P* < 0.001), lymph-node metastasis (*P* < 0.001), margin status (*P* = 0.002), tumor differentiation (*P* = 0.023), perineural invasion (*P* = 0.024), vascular invasion (*P* = 0.035), and EHBD resection (*P* = 0.008) were significantly associated with the OS. To further investigate whether EHBD resection was independently associated with OS, multivariable analyses were performed. The results showed that T category (*P* < 0.001, HR = 3.610, 95% CI 2.277–5.724), lymph-node metastasis (*P* < 0.001, HR = 2.452, 95% CI 1.606–3.750), margin status (*P* = 0.008, HR = 1.895, 95% CI 1.183–3.037), EHBD resection (*P* < 0.001, HR = 0.503, 95% CI 0.349–0.725) and the pre-operative CA19-9 level (*P* = 0.030, HR = 1.476, 95% CI 1.038–2.098) were identified as independent prognostic factors for OS.

**Table 2.** Univariate and multivariable analysis of advanced GBC associated with OS

Variable	Before PS matching				After PS matching			
	Univariate analysis	Multivariate analysis			Univariate analysis	Multivariate analysis		
	*P*	HR	95% CI	*P*	*P*	HR	95% CI	*P*
Sex (males vs. females)	0.343				0.949			
Age (≤60 vs. >60 years)	0.448				0.574			
Cholelithiasis (yes vs. no)	0.310				0.606			
CA19-9 (≤37 vs. >37 U/mL)	<0.001	1.476	1.038–2.098	0.030	<0.001	1.560	1.015–2.397	0.043
T category (T2 vs. T3/T4)	<0.001	3.610	2.277–5.724	<0.001	<0.001	2.029	1.413–2.914	<0.001
Lymph-node metastasis (yes vs. no)	<0.001	2.452	1.606–3.750	<0.001	<0.001	2.259	1.343–3.801	0.002
Margin status (R0 vs. R1)	0.002	1.895	1.183–3.037	0.008	<0.001	1.952	1.154–3.302	0.013
Tumor differentiation (poorly vs. well/moderately)	0.023	0.774	0.537–1.115	0.169	0.010	0.670	0.429–1.046	0.078
Perineural invasion (yes vs. no)	0.024	0.781	0.485–1.257	0.309	0.042	0.678	0.391–1.173	0.164
Vascular invasion (yes vs. no)	0.035	1.292	0.829–2.013	0.257	0.195			
EHBD resection (yes vs. no)	0.008	0.503	0.349–0.725	<0.001	0.032	0.588	0.388–0.891	0.012
Post-operative complications (yes vs. no)	0.127				0.919			
Post-operative chemotherapy (yes vs. no)	0.219				0.181			

GBC, gallbladder carcinoma; OS, overall survival; PS matching, propensity score matching; EHBD, extra-hepatic bile duct; HR, hazard ratio; 95% CI, 95% confidence interval.

### Propensity score matching analysis

To reduce the selection bias inherent in retrospective observational study, a propensity score matching analysis was performed (Table 1). The propensity score was calculated with pre-operative factors and independent prognostic factors including age, sex, body mass index, pre-operative CA19-9 level, T category, lymph-node metastasis and margin status.

After propensity score matching, the pre-operative ALT level in the EHBD resection group was significantly higher than that in the non-EHBD resection group (24 vs. 17 IU/L, *P* = 0.016). Meanwhile, the pre-operative TB level, perineural invasion rate, post-operative complications rate, the number of lymph nodes retrieved and the number of positive lymph nodes were still significantly higher in the EHBD resection group than in the non-EHBD resection group (all *P* values <0.05).

**Figure 2.**
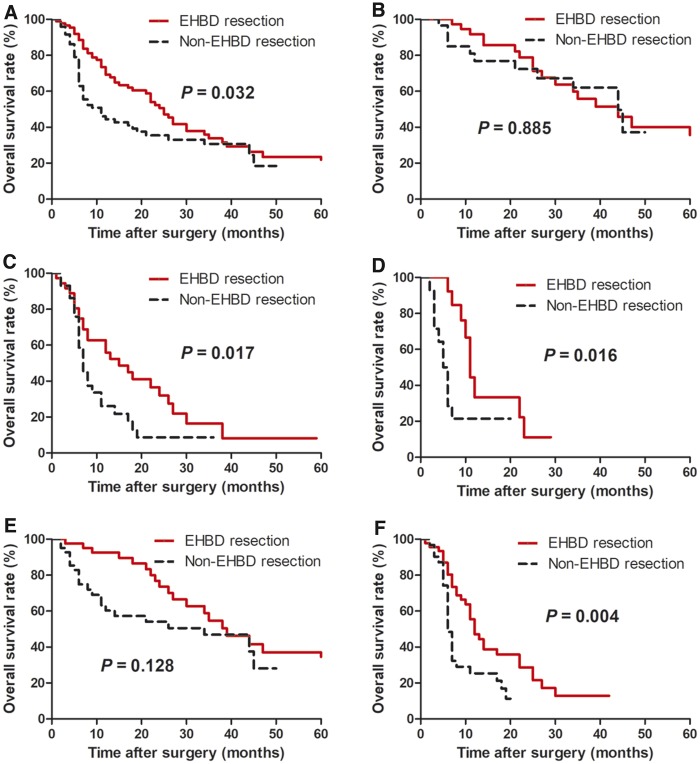
Kaplan–Meier OS analysis of patients with advanced GBC undergoing EHBD resection after propensity score matching. (**A**) The entire cohort; (**B**) the patients with T2 lesion; (**C**) the patients with T3 lesion; (**D**) the patients with T4 lesion; (**E**) the patients without lymph-node metastasis; (**F**) the patients with lymph-node metastasis. OS, overall survival; GBC, gallbladder carcinoma; EHBD, extra-hepatic bile duct.

Patients who underwent EHBD resection still had a longer median OS than those without EHBD resection (25 vs. 11 months, *P* = 0.032, Figure 2A). Similarly, the EHBD resection group achieved better median OS than the non-EHBD resection group in GBC patients with T3 lesion (15 vs. 7 months, *P* = 0.017; Figure 2C), T4 lesion (11 vs. 5.5 months, *P* = 0.016; Figure 2D) and lymph-node metastasis (12 vs. 6 months, *P* = 0.004; Figure 2F). However, no survival benefit of EHBD resection were observed in GBC patients with T2 lesion (44 vs. 44 months, *P* = 0.885; Figure 2B) or without lymph-node metastasis (39 vs. 34 months, *P* = 0.128; Figure 2E).

After propensity score matching, the univariate analysis results still showed that CA19-9 level (*P* < 0.001), T category (*P* < 0.001), lymph-node metastasis (*P* < 0.001), margin status (*P* < 0.001), tumor differentiation (*P* = 0.010), perineural invasion (*P* = 0.042) and EHBD resection (*P* = 0.032) were significantly associated with OS. However, vascular invasion was not statistically significant after propensity score matching (*P* = 0.195). These five factors—T category (*P* < 0.001, HR = 2.029, 95% CI 1.413–2.914), lymph-node metastasis (*P* = 0.002, HR = 2.259, 95% CI 1.343–3.801), margin status (*P* = 0.013, HR = 1.952, 95% CI 1.154–3.302), EHBD resection (*P* = 0.012, HR = 0.588, 95% CI 0.388–0.891) and pre-operative CA19-9 level (*P* = 0.043, HR = 1.560, 95% CI 1.015–2.397)—were still significant independent prognostic factors.

## Discussion

According to previous reports, the oncological benefit of prophylactic resection combined EHBD resection for locally advanced GBC without EHBD involvement is controversial [12, 21, 22]. In the current study, we analysed the clinical impact of combined EHBD resection for advanced GBC. Our findings showed that, for GBC patients with T3 lesion, T4 lesion and lymph-node metastasis, combined EHBD resection is associated with improved OS. Prophylactic resection of the EHBD in GBC patients with T2 lesion or without lymph-node metastasis was a futile exercise. Meanwhile, T category, lymph-node metastasis, margin status, pre-operative CA19-9 level and EHBD resection were independent prognostic factors for advanced GBC.

GBC partially lacks serosa and has a relatively thin proper muscle layer, which leads to the propensity for local invasion and distant metastases [23]. Thus, GBC is a devastating malignant neoplasm with a poor prognosis, with an estimated 5-year survival rate of <20% [24]. Receiving R0 resection is the only chance for GBC patients to improve survival. The extent of curative surgery for GBC remains controversial. In particular, whether EHBD resection should be routinely performed for advanced GBC remains unclear. While some surgeons recommend routine resection of the EHBD for GBC [7, 8], others focus on the poor survival benefits and post-EHBD resection complications [25–27].

Several institutions have advocated routine EHBD resection for GBC [7, 8, 28, 29]. Chikamoto *et al*. [13] found that, in 20% (3/15) of GBC patients, clusters of cancer cells were identified in submucosal lymph vessels of the EHBD by special immunohistochemical staining; however, this invasion failed to be detected by conventional hematoxylin–eosin staining. Shimizu *et al.* [8] conducted routine resection of EHBD in patients with locally advanced GBC and found that 27.2% (9/33) of patients suffered microvessel invasion, including lymphatic, venous and perineural invasions around the EHBD. These results indicate that routine resection of EHBD may be of value in curative resection for advanced GBC for a radical intent. Furthermore, complete lymph-node dissection in the hepatoduodenal ligament may compromise the EHBD owing to devascularization. Thus, Sakamoto *et al*. [7] advocated for routine EHBD resection to completely eradicate the regional nodes and avoid EHBD ischemia. In GBC patients with the presence of perineural invasion, combined EHBD resection showed significantly better survival than EHBD-preserved resection [7]. However, the precise pre-operative assessment of perineural invasion was difficult, strongly suggesting that EHBD resection should be performed in all patients with advanced GBC. In our series, 19.7% of advanced GBC patients had perineural invasion, and perineural invasion was demonstrated as an independent prognostic factor. In addition, EHBD resection was associated with improved OS, perhaps due to thorough dissection of the lymph nodes and perineural invasion in the connective tissues.

As stated in the literature, EHBD resection is necessary to achieve complete lymph-node dissection. For the number of retrieved lymph nodes, EHBD resection was found to be associated with improved lymph-nodal dissection [30]. In contrast, Sakamoto *et al.* [7] reported that the mean number of dissected lymph nodes was comparable between the EHBD resection group and the non-EHBD resection group (13 vs. 10, *P* = 0.47). In our study, we found that the median number of lymph nodes retrieved in the EHBD resection group was more than that in the non-EHBD resection group (6 vs. 4, *P* < 0.001). Furthermore, the median number of positive lymph nodes in the EHBD resection group was also larger than that in the EHBD resection group (3 vs. 2, *P* = 0.006). In the EHBD resection group, 13.8% of patients received pancreatoduodenectomy, whereas no patients received pancreatoduodenectomy in the non-EHBD resection group. Therefore, infiltration of the lower bile duct is more likely to happen and the possibility of lymph-node involvement is more likely to occur in the EHBD resection group, which might be another reason why the numbers of retrieved lymph nodes or positive lymph nodes were larger in the EHBD resection group than those in the non-EHBD resection group.

Although routine EHBD resection for advanced GBC may have advantages in theory, the survival benefit of EHBD resection is controversial. A recent survey of the Japanese Society of Biliary Surgery reported no difference in OS between the EHBD resection and non-EHBD resection groups in GBC patients with lymph-node metastasis and T2, T3 or T4 lesion, and they concluded that EHBD resection may therefore be unnecessary in advanced GBC without infiltration of the cystic duct [31]. Gani *et al.* [12] conducted a multi-institutional study including 449 GBC patients and found that EHBD resection was not associated with improved long-term outcomes. In contrast, Kohya *et al.* [32] reported substantial improvement in survival of patients undergoing EHBD resection compared with that of patients without EHBD resection. Sakamoto *et al.* [7] found that EHBD resection is associated with prolonged OS when perineural invasion exists, even in patients without biliary infiltration. In the current study, the median OS of the EHBD resection group was longer than that of the non-EHBD resection group (25 vs. 11 months, *P* = 0.008). In particular, the median OS was significantly longer in the EHBD resection group for GBC patients with T3 lesion (15 vs. 7 months, *P* = 0.002), T4 lesion (11 vs. 6 months, *P* = 0.021) and lymph-node metastasis (12 vs. 7 months, *P* < 0.001). Our study showed that EHBD resection should be performed selectively and is associated with improved OS.

Although EHBD resection may be associated with improved OS of GBC, the procedure resulted in increased post-operative morbidity associated with bilioenteric anastomosis, such as bile leakage. Fuks *et al.* [25] reported a multi-centric database which demonstrates that EHBD resection was associated with increased post-operative morbidity. Araida *et al.* [31] reported a significantly higher morbidity in the EHBD resection group vs. that in the non-EHBD resection group (21.7% vs. 10%, *P* < 0.001). Moreover, patients undergoing EHBD resection showed significantly longer operation time and post-operative hospital stay than those with EHBD resection, which were associated with a higher occurrence of complications. Our results were similar to the findings of previous reports. In our study, the EHBD resection group suffered more post-operative complications (33.3% vs. 21.4%, *P* = 0.046). In particular, the frequency of bile leakage in the EHBD group was higher than that in the non-EHBD resection group (9.2% vs. 4.0%, *P* = 0.117), although it did not reach statistical significance. However, these disadvantages of EHBD resection will be overcome by improvements in surgical technique, anesthesia and post-operative care.

Although propensity score matching analysis was performed in our study, a prospective, multi-institutional study is warranted to objectively evaluate the clinical significance of EHBD resection during curative surgery for advanced GBC.

## Conclusions

Combined EHBD resection is justified for advanced GBC patients with T3 lesion, T4 lesion and lymph-node metastasis. Considering the high post-operative morbidity, EHBD resection for advanced GBC patients should be performed by high-volume experienced surgeons in highly specialized medical centers.

## Authors’ contribution

J.K.W. and W.J.M. contributed to data acquisition and drafted the manuscript. Z.R.W., Q.Y., H.J.H., and F.L. revised the manuscript. F.Y.L. contributed to the study design and revision of the manuscript. All authors have approved this manuscript for publication.

## Funding

This work was supported by the grant from the Science & Technology Support Project of Sichuan Province (No. 2018JY0019).
